# Preferential Selection of Low-Frequency, Lipopolysaccharide-Modified, Colistin-Resistant Mutants with a Combination of Antimicrobials in Acinetobacter baumannii

**DOI:** 10.1128/spectrum.01928-22

**Published:** 2022-09-29

**Authors:** Go Kamoshida, Noriteru Yamada, Tomoka Nakamura, Daiki Yamaguchi, Daichi Kai, Maho Yamashita, Chiaki Hayashi, Nana Kanda, Moe Sakaguchi, Hitoshi Morimoto, Teppei Sawada, Tomoko Okada, Yuki Kaya, Norihiko Takemoto, Kinnosuke Yahiro

**Affiliations:** a Department of Microbiology and Infection Control Sciences, Kyoto Pharmaceutical University, Kyoto, Japan; b Pathogenic Microbe Laboratory, Research Institute, National Center for Global Health and Medicine, Tokyo, Japan; Università Roma Tre

**Keywords:** *Acinetobacter baumannii*, LPS, colistin, *lpxACD*, meropenem, *pmrAB*

## Abstract

Colistin, which targets lipopolysaccharide (LPS), is used as a last-resort drug against severe infections caused by drug-resistant Acinetobacter baumannii. However, A. baumannii possesses two colistin-resistance mechanisms. LPS modification caused by mutations in *pmrAB* genes is often observed in clinical isolates of multidrug-resistant Gram-negative pathogens. In addition to LPS modification, A. baumannii has a unique colistin resistance mechanism, a complete loss of LPS due to mutations in the *lpxACD* genes, which are involved in LPS biosynthesis. This study aimed to elucidate the detailed mechanism of the emergence of colistin-resistant A. baumannii using strains with the same genetic background. Various colistin-resistant strains were generated experimentally using colistin alone and in combination with other antimicrobials, such as meropenem and ciprofloxacin, and the mutation spectrum was analyzed. *In vitro* selection of A. baumannii in the presence of colistin led to the emergence of strains harboring mutations in *lpxACD* genes, resulting in LPS-deficient colistin-resistant strains. However, combination of colistin with other antimicrobials led to the selection of *pmrAB* mutant strains, resulting in strains with modified LPS (LPS-modified strains). Further, the LPS-deficient strains showed decreased fitness and increased susceptibility to many antibiotics and disinfectants. As LPS-deficient strains have a higher biological cost than LPS-modified strains, our findings suggested that *pmrAB* mutants are more likely to be isolated in clinical settings. We provide novel insights into the mechanisms of resistance to colistin and provide substantial solutions along with precautions for facilitating current research and treatment of colistin-resistant A. baumannii infections.

**IMPORTANCE** Acinetobacter baumannii has developed resistance to various antimicrobial drugs, and its drug-resistant strains cause nosocomial infections. Controlling these infections has become a global clinical challenge. Carbapenem antibiotics are the frontline treatment drugs for infectious diseases caused by A. baumannii. For patients with infections caused by carbapenem-resistant A. baumannii, colistin-based therapy is often the only treatment option. However, A. baumannii readily acquires resistance to colistin. Many patients infected with colistin-resistant A. baumannii undergo colistin treatment before isolation of the colistin-resistant strain, and it is hypothesized that colistin resistance predominantly emerges under selective pressure during colistin therapy. Although the concomitant use of colistin and carbapenems has been reported to have a synergistic effect *in vitro* against carbapenem-resistant A. baumannii strains, our observations strongly suggest the need for attention to the emergence of strains with a modified lipopolysaccharide during treatment.

## INTRODUCTION

Acinetobacter baumannii is a Gram-negative bacterium widely distributed in nature. Although A. baumannii is detected on the skin of healthy individuals and is generally harmless, it can cause opportunistic infections in immunocompromised hosts. The ability of this pathogen to survive in harsh environments, such as under desiccated conditions, enables its survival in hospital facilities and on medical devices, causing a spectrum of infectious diseases of the respiratory tract, bloodstream, urinary tract, surgical sites, and wounds (1, 2). Although health care professionals are paying attention to A. baumannii infections, this pathogen is responsible for a vast array of nosocomial infections, and the difficulty of controlling these infections remains a problem of global importance. One reason for this difficulty is its ability to readily acquire antimicrobial resistance. Acinetobacter baumannii exhibits multiple mechanisms of antimicrobial resistance, including production of several intrinsic and acquired β-lactamase enzymes, efflux pumps that prevent accumulation of antimicrobials, and acquired resistance through plasmids carrying resistance genes. Consequently, pandrug-resistant A. baumannii is a growing and alarming global health concern (2–8). Although carbapenem antibiotics are used as frontline treatment for A. baumannii, carbapenem-resistance is widely observed in several international clones, and the WHO classifies A. baumannii as a bacterium that requires new antibiotic development.

Currently, colistin-based therapy is the most efficient treatment option for patients with infections caused by carbapenem-resistant A. baumannii (9–13). Colistin is classified as a cationic amphiphilic polypeptide antibacterial drug that interacts with the lipid A component of the outer membrane lipopolysaccharide (LPS) and disrupts bacterial membrane integrity, ultimately causing cell death. Colistin is used as a last resort drug to combat infections caused by severe multidrug-resistant (MDR) A. baumannii and other MDR Gram-negative bacteria (14–17). The increased use of colistin in human pharmacotherapy has led to an increase in colistin-resistant bacterial strains, which has caused major concern worldwide due to the lack of antimicrobials available for treatment of these resistant strains (18, 19). In particular, the high frequency of acquisition of colistin resistance by A. baumannii is concerning. Epidemiological surveillance shows that colistin-resistant strains of A. baumannii are not phylogenetically restricted and most colistin-resistant clinical isolates have acquired mutations in chromosomal genes related to colistin resistance (20–24).

The general mechanism of resistance to colistin is due to modification of LPS with phosphoethanolamine, which reduces the electrostatic interaction between colistin and LPS (14, 17). Acinetobacter baumannii has an endogenous phosphoethanolamine transferase (PmrC) that is regulated by the two-component regulatory system PmrAB, and it is the main enzyme responsible for LPS modification and colistin resistance (25, 26). Notably, A. baumannii has another colistin resistance mechanism, which is the complete loss of LPS due to mutations in *lpxACD* genes, which are responsible for LPS biosynthesis. LPS is thought to be essential for the survival of Gram-negative bacteria; therefore, inhibition of its synthesis is a target for novel antimicrobial agents (27–31). However, a few species, such as Neisseria meningitidis, Moraxella catarrhalis, A. baumannii, and Acinetobacter nosocomialis can survive with complete loss of LPS (32–34). The emergence of *lpxACD* mutants of A. baumannii under significant outer membrane stress caused by polymyxin antibiotics in both laboratory and clinical settings has been reported (29, 35, 36). However, most clinically isolated colistin-resistant strains exhibit LPS modification (referred to here as LPS-modified strains) due to the *pmrAB* mutations (37, 38). Additionally, some A. baumannii strains have been reported to exhibit a colistin-dependent transient phenotype for survival under colistin pressure (39, 40). The detailed mechanism through which A. baumannii acquires colistin resistance remains unknown. In particular, fundamental data representing the spectrum of mutation types have not been obtained.

## RESULTS

### Mutation spectrum of colistin-resistant strains obtained in the laboratory and clinical settings.

**FIG 1:**
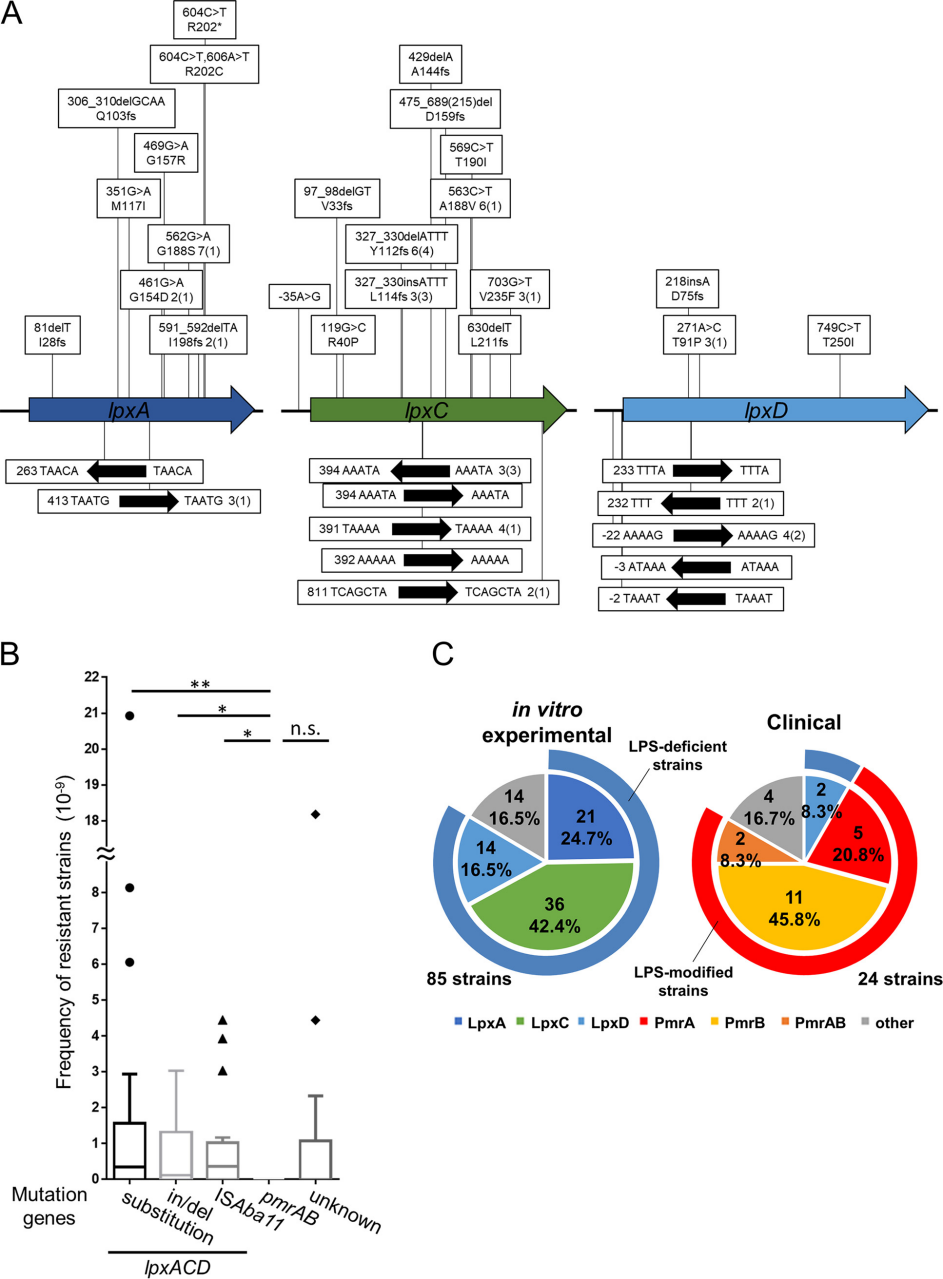
Mutation sites of colistin-resistant A. baumannii strains in laboratory and clinical settings. (A) Mutation site analysis of *in vitro* experimental laboratory-isolated strains. A. baumannii ATCC 19606 strains were isolated by direct plating on LB agar plates containing 5× MIC of colistin (10 μg/mL) to select resistant strains. Sanger sequencing was performed on the genomic regions containing the *lpxACD* genes. In the boxes above the genes, base changes are shown on top, while amino acid changes are shown below. The numerical values are the numbers of isolated strains, and numbers in parentheses are the numbers of independent events. The black arrows indicate IS*Aba11*, and repetitive sequences are shown. (B) Frequency analysis of occurrence for each mutation. Data are shown in a box plot; 18 independent assays were compared by one-way ANOVA. n.s., not significant; **, *P* < 0.01; *, *P* < 0.05. Of the 110 colistin-resistant strains, 28 could not be sequenced because of overlapping colonies. (C) Pie chart showing mutation rates. Data for isolated *in vitro* experimental strains are on the left, and the results of NCBI BioSample analysis are on the right.

To analyze how A. baumannii acquires resistance to colistin, we used A. baumannii type strain ATCC 19606 as a model strain. The bacterium adopts two different resistance mechanisms: loss-of-function mutations in genes involved in LPS biosynthesis (*lpxACD*), resulting in LPS-deficient strains, and gain-of-function mutations in *pmrAB*, which regulate the expression of the LPS modification enzyme (PmrC), resulting in LPS-modified strains (1, 17, 28). To assess frequently occurring mutations in A. baumannii, we independently cultured 16 single colonies; aliquots were spread on plates supplemented with 10 μg/mL (5× MIC) of colistin, and the frequency of mutation types was analyzed. Eighty-five of the isolated colistin-resistant mutants of A. baumannii ATCC 19606 were subjected to PCR amplification of the *lpxA*, *lpxC*, *lpxD*, *pmrA*, and *pmrB* genes. The PCR products were sequenced to determine mutations that lead to colistin resistance (Fig. 1A). To compare the occurrence of each mutation, the frequency of each mutation was calculated by dividing the number of colonies with each mutation by the CFU obtained on colistin-containing plates. The frequencies of colistin-resistant strains harboring substitutions, short indels, and insertion of IS*Aba11* in *lpx* genes were similar, but strains harboring mutations in *pmrAB* were not detected (Fig. 1B). Mutations in *lpxA*, *lpxC*, and *lpxD* genes were detected in 21, 36, and 14 colistin-resistant strains, respectively, and the locations of these mutations were spread broadly throughout the genes (Fig. 1A; also, see Table S1 in the supplemental material). In 34% (24 strains) of the colistin-resistant strains, IS*Aba11* was inserted into the *lpx* genes, disrupting each gene. Mutations (KL068) or IS*Aba11* insertions (KL003, -045, -070, and -083) upstream of the *lpx* genes were also identified, and we confirmed that these mutations downregulated mRNA expression of downstream *lpx* genes (Fig. S1). In summary, we found nonsynonymous mutations in the *lpx* genes of 71 colistin-resistant A. baumannii strains, but none harbored mutations in *pmrAB* (Fig. 1A; Tables S1 and 2).

The MICs of colistin for most strains with amino acid substitutions in LpxACD were similar to those determined for strains with frameshift mutations. Only strain KL052, which harbored a mutation leading to a T91P substitution in LpxD, exhibited a low MIC of colistin (Table S3). The observed mutations in *lpxACD* genes comprised nonsynonymous substitutions, short indels, and transposition of the insertion sequence IS*Aba11* and possibly resulted in loss of function of enzymes involved in lipid A synthesis. In the remaining 14 colistin-resistant strains, no mutations were detected in either *lpxACD* or *pmrAB*, and the resistance mechanism remains unknown. In total, 151 colistin-resistant strains of A. baumannii ATCC 19606 were identified, including the strains that were not sequenced (66 strains). Among these, only one strain showed growth in the presence of colistin (5× MIC) and meropenem (1/5× MIC), and subsequent sequence analysis revealed mutations in *pmrB* (KL148; *pmrB* 697C→T, P233S). These results indicate that the colistin-resistant mutations in A. baumannii are more likely to occur in *lpxACD* genes than in *pmrAB* genes *in vitro*.

Next, we performed *in silico* analysis using the NCBI BioSample database to understand how clinical isolates of A. baumannii acquire resistance to colistin. Among the 24 clinically isolated colistin-resistant strains analyzed, only two harbored an amino acid substitution in LpxD, and 18 harbored an amino acid substitution in PmrAB (Table S4). Thus, in contrast to our *in vitro* experimental observations that indicated a higher frequency of LPS-deficient strains, most colistin-resistant clinical isolates were PmrAB mutants and therefore had LPS modifications (Fig. 1C).

### Change in colistin-resistant mutation type due to combination with meropenem.

**FIG 2:**
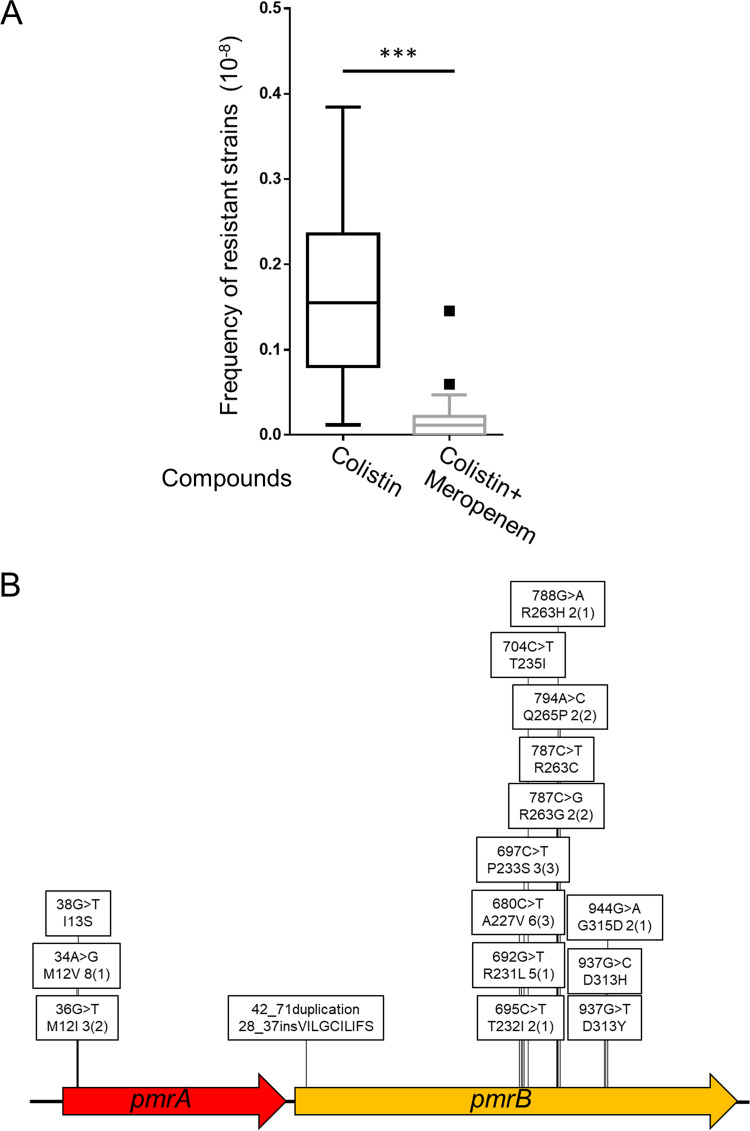
Frequency of emergence of colistin-resistant A. baumannii (type strain, ATCC 19606) strains and changes in mutation tendency in combination with meropenem. (A) The frequency of colistin resistance was analyzed by plating 0.2 mL of ATCC 19606 culture (OD_600_ of 1.0) on LB agar containing 5× MIC (10 μg/mL) of colistin for 24 h at 37°C. The frequency of resistance to colistin, when combined with 1/5× MIC (0.1 μg/mL) of meropenem, was determined by plating 0.1 mL of ATCC 19606 culture (OD_600_ of 10) on LB agar containing 10 μg/mL of colistin and 0.1 μg/mL of meropenem. Data are shown in a box plot; 20 independent assays were compared by the Mann-Whitney *U* test. ***, *P* < 0.001. (B) Mutation site analysis of colistin-meropenem-resistant ATCC 19606 strains isolated by direct plating on LB agar plates containing 5× MIC (10 μg/mL) of colistin and 1/5× MIC (0.1 μg/mL) of meropenem. Sanger sequencing was performed on the genomic regions containing the *pmrAB* genes. In the boxes, base changes are shown on top, while amino acid changes are shown below. The numerical values are the numbers of isolated strains, and numbers in parentheses are the numbers of independent events.

The colistin resistance mutations differed between *in vitro* experimental and clinically isolated strains of A. baumannii. In clinical practice, colistin is often used with other antibiotics, such as carbapenem, after failure of first-line drugs (22, 23). In cases where the use of colistin is necessary, these first-line drugs may still act at sub-MICs, as a withdrawal period cannot be established. Therefore, we investigated colistin-resistant mutants obtained in combination with meropenem, which is commonly used to treat A. baumannii infections. The frequency of colistin resistance was determined when A. baumannii was treated with a combination of colistin (5× MIC) and meropenem (sub-MIC [1/5× MIC]). Although no growth disparity was observed following treatment with 1/5× MIC of meropenem alone, combinational treatment with meropenem and colistin decreased the frequency of colistin-resistant colonies to approximately 1/8 of that determined with colistin alone (Fig. 2A). We isolated 41 colistin-meropenem-resistant A. baumannii strains, and their genetic mutations were analyzed. As shown in Fig. 2B, nonsynonymous point mutations or 30-bp duplications resulting in 10 amino acid insertions in *pmrAB* genes were identified in all 41 colistin-meropenem-resistant strains (Table S1). Mutations in *pmrAB* genes were restricted to several sites, and most of these sites were identical to those observed in clinical isolates (Fig. 2B and Table S5) (41, 42). Further, the *lpxACD* genes were analyzed in 21 of the 41 colistin-meropenem-resistant strains, but no mutations were observed (Table S5). Thus, although the dose of meropenem was quite low compared to that used in clinical settings, the addition of meropenem resulted in the preferential selection of *pmrAB* mutants possibly harboring modified LPS.

### Analysis of the mutation types of colistin resistance in combination with ciprofloxacin and in drug-resistant strains.

**FIG 3:**
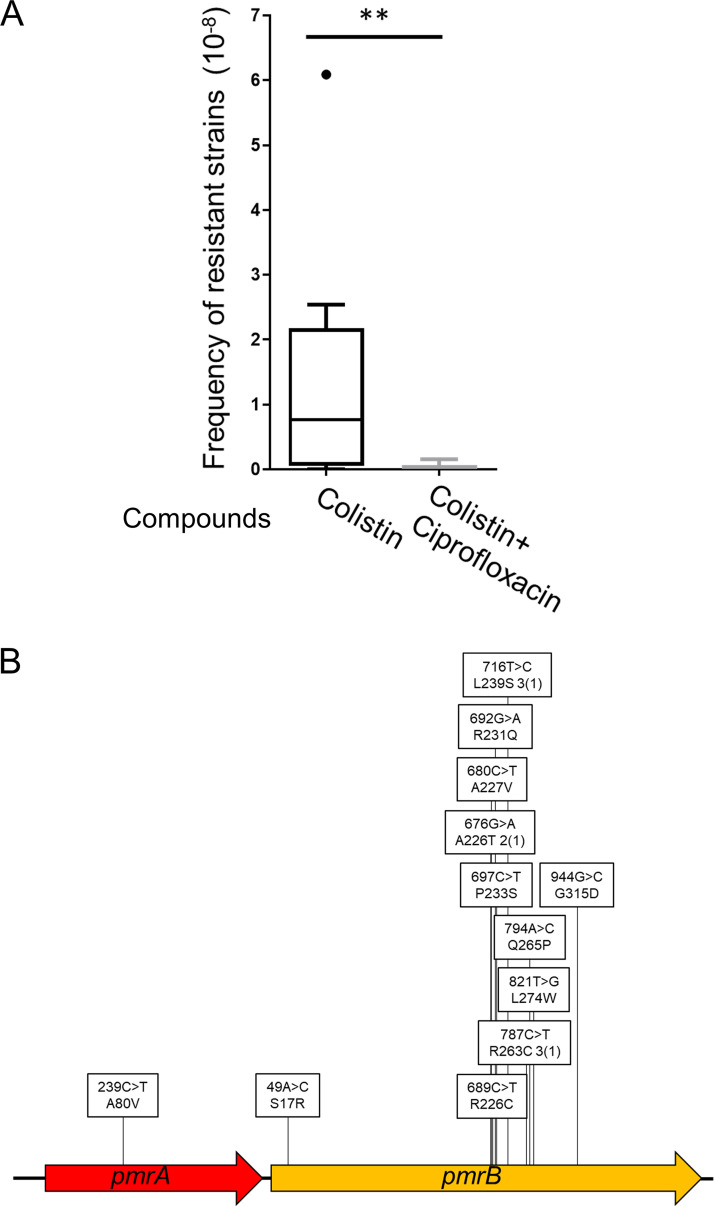
Frequency of emergence of colistin-resistant A. baumannii ATCC 19606 strains and altered mutation tendencies in combination with ciprofloxacin. (A) The frequency of colistin resistance was analyzed by plating 0.2 mL culture (OD_600_ of 1.0) on LB agar containing 5× MIC (10 μg/mL) of colistin and incubating for 24 h at 37°C. The frequency of colistin resistance in combination with ciprofloxacin was determined by plating 0.1 mL culture (OD_600_ of 10) on LB agar containing 10 μg/mL of colistin and 0.4 μg/mL (1/5× MIC) of ciprofloxacin. Data are shown as box plots (*n *= 10 independent assays). Comparisons were made using the Mann-Whitney *U* test. **, *P* < 0.01. (B) Mutation site analysis of colistin-ciprofloxacin-resistant ATCC 19606 strains isolated by direct plating on LB agar containing 10 μg/mL colistin and 0.4 μg/mL of ciprofloxacin. Sanger sequencing was performed on genomic regions containing the *pmrAB* genes. In the boxes, base changes are shown on top, while amino acid changes are shown below. The numerical values are the numbers of isolated strains, and numbers in parentheses are the numbers of independent events.

We next investigated the frequency of colistin resistance and the mutation spectrum using a combination of colistin (5× MIC) and ciprofloxacin (1/5× MIC). Ciprofloxacin is used against resistant bacterial strains, and its mechanism of action differs from that of meropenem by targeting DNA (43). Combination with ciprofloxacin reduced the frequency of colistin resistance (Fig. 3A), and all 17 colistin-ciprofloxacin-resistant strains harbored mutations in the *pmrAB* genes (Fig. 3B and Table S6).

**FIG 4:**
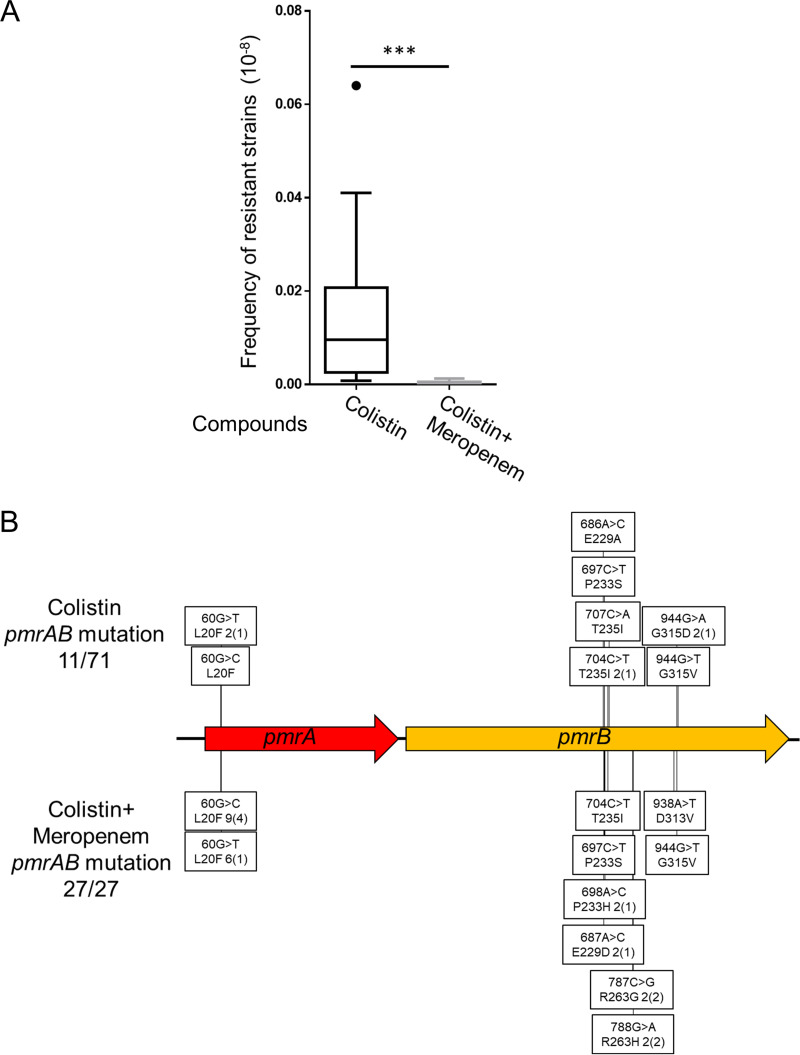
Frequency of emergence of colistin-resistant A. baumannii (ATCC BAA-1605) strains and change in mutation tendency in combination with meropenem. (A) The frequency of colistin resistance was analyzed by plating 0.1 mL culture (OD_600_ 1.0) on LB agar containing 5× MIC (10 μg/mL) of colistin for 24 h at 37°C. The frequency of colistin resistance in combination with 1/5× MIC (2 μg/mL) of meropenem was determined by plating 0.1 mL culture (OD_600_ of 25) on LB agar containing 10 μg/mL of colistin and 2 μg/mL of meropenem. Data are shown as a box plot; 20 independent assays were compared by the Mann-Whitney *U* test. ***, *P* < 0.001. (B) Mutation site analysis of colistin- and colistin-meropenem-resistant ATCC BAA-1605 strains. Sanger sequencing of the genomic regions containing the *pmrAB* genes was performed. Mutations in colistin-resistant strains and colistin-meropenem-resistant strains are shown above and below the genes, respectively. In the boxes, base changes are shown on top, while amino acid changes are shown below. The numerical values are the numbers of isolated strains, and numbers in parentheses are the numbers of independent events.

Furthermore, to test whether these observations are accurate in a clinical multidrug-resistant strain of A. baumannii, we analyzed the frequency of colistin-resistant mutants in the strain ATCC BAA-1605. It was originally resistant to ceftazidime, gentamicin, ticarcillin, piperacillin, aztreonam, cefepime, ciprofloxacin, imipenem, and meropenem; the MIC of meropenem was 10 μg/mL. Additionally, this strain was sensitive to amikacin and tobramycin (44, 45). Among the 71 colistin-resistant strains obtained in the absence of meropenem, 11 (approximately 15%) strains showed growth in the presence of both colistin (5× MIC) and meropenem (1/5× MIC). Analysis of the related genes showed that these strains harbored mutations in *pmrAB* (Fig. 4B and Table S7). Sequence analysis of a proportion of the remaining 60 strains that did not grow on the plate containing colistin and meropenem revealed that these strains harbored mutations in *lpxACD* genes but not in *pmrAB* genes (data not shown). Interestingly, although the ATCC BAA-1605 strain lacks IS*Aba11* in its genome, PCR analysis targeting *lpx* genes in colistin-resistant strains resulted in the amplification of fragments larger than the expected size in 33 strains (*lpxA* [2 strains], *lpxC* [28 strains], and *lpxD* [3 strains]) among the 71 strains (46%) (Table S8). Sequence analysis of some of these fragments demonstrated that the change in size was due to the insertion of IS*Aba1* (data not shown). The frequency of colistin-resistant mutants of ATCC BAA-1605 in the absence of meropenem was approximately 10-fold lower than that of ATCC 19606. The combination with 1/5× MIC of meropenem reduced the frequency of colistin resistance in ATCC BAA-1605 (Fig. 4A). As observed for ATCC 19606, all 27 ATCC BAA-1605 colistin-resistant strains identified in combination with meropenem were *pmrAB* mutants (Fig. 4B and Table S7). Thus, preferential selection of *pmrAB* mutants by the combined use of colistin with sub-MIC of antimicrobials occurred in laboratory and clinical drug-resistant strains.

### Susceptibility to other antibiotics and phenotype in colistin-resistant mutants.

The LPS-deficient mutant of A. baumannii is more susceptible to some antibiotics, such as β-lactams, than the wild-type strain (35, 36, 40). Representative strains of each mutation were selected to examine colistin susceptibility as a function of resistance mutations. To exclude the possibility of contamination with genetically different strains during the isolation of representative strains from a large population, single colonies were isolated on plates supplemented with 10 μg/mL (5 × MIC) colistin. For example, KL001 was selected as a representative *lpxA* mutant and subjected to single-colony isolation, and the resultant monoclonal strain was named KL001S. The analysis of the whole-genome sequence obtained by next-generation sequencing confirmed that the *lpxACD* or *pmrAB* mutation was the sole mutation, suggesting that they are responsible for colistin resistance. Three strains (KL001S, KL037S, and KL055S) had mutations in the *lpxACD* genes responsible for LPS biosynthesis. The MIC of colistin for these *lpx* mutant strains was 128 μg/mL. In *pmrAB* mutant strains (CM022S [*pmrA*] and CM012S [*pmrB*]), the MIC of colistin was 1,024 μg/mL (Table 1). Next, we examined susceptibility to other antibiotics viz meropenem, amikacin, ciprofloxacin, and tigecycline. As expected, the MICs of these antibiotics in *pmrAB* mutant strains were similar to those determined in the wild-type strain. In contrast, the MICs of meropenem, amikacin, and ciprofloxacin decreased substantially in *lpxACD* mutant strains; however, the MIC of tigecycline was similar to that in the wild type or *pmrAB* mutants (Table 2).

**TABLE 1:** Gene mutation analysis, MIC of colistin, and LAL assay results for representative colistin-resistant A. baumannii strains

Mutated gene	Strain	Mutation	Colistin MIC (μg/mL)	Endotoxin concn (EU/mL)^*a*^
None (wild type)	ATCC 19606		2	9.2 × 10^3^ ± 6.0 × 10^3^
*lpxA*	KL001S	591_592delTA: I198fs(I198*)	128	<0.1
*lpxC*	KL037S	475_689(215)del: D159fs(D159C)D161*	128	<0.1
*lpxD*	KL055S	218insA: D75fs(D75R)N76*	128	<0.1
*pmrA*	CM022S	36G>T: M12I	1024	17 × 10^3^ ± 8.7 × 10^3^
*pmrB*	CM012S	697C>T: P233S	1024	13 × 10^3^ ± 9.8 × 10^3^

a Determined by the LAL assay at an OD of 0.1. EU, endotoxin units. Values are means ± SD.

**TABLE 2:** MICs of antimicrobials and disinfectants against representative colistin-resistant A. baumannii strains

Agent (MIC units)	MIC for:					
	Wild-type ATCC 19606	*lpxACD* mutants			*pmrAB* mutants	
		KL001S	KL037S	KL055S	CM022S	CM012S
Colistin (μg/mL)	2	128	128	128	1,024	1,024
Meropenem (μg/mL)	0.5	<0.016	<0.016	<0.016	0.5	0.25
Amikacin (μg/mL)	16	1	0.5	0.5	16	16
Ciprofloxacin (μg/mL)	2	0.125	0.125	0.0625	1	1
Tigecycline (μg/mL)	0.5	0.5	0.5	0.5	0.5	0.5
Ethanol (%)	3.125	3.125	3.125	3.125	3.125	3.125
H_2_O_2_ (%)	0.003	0.003	0.003	0.003	0.003	0.003
SDS (%)	0.5	0.004	0.004	0.004	0.25	0.25
Benzalkonium (%)	0.0006	0.00016	0.00016	0.000078	0.0006	0.0006

**FIG 5:**
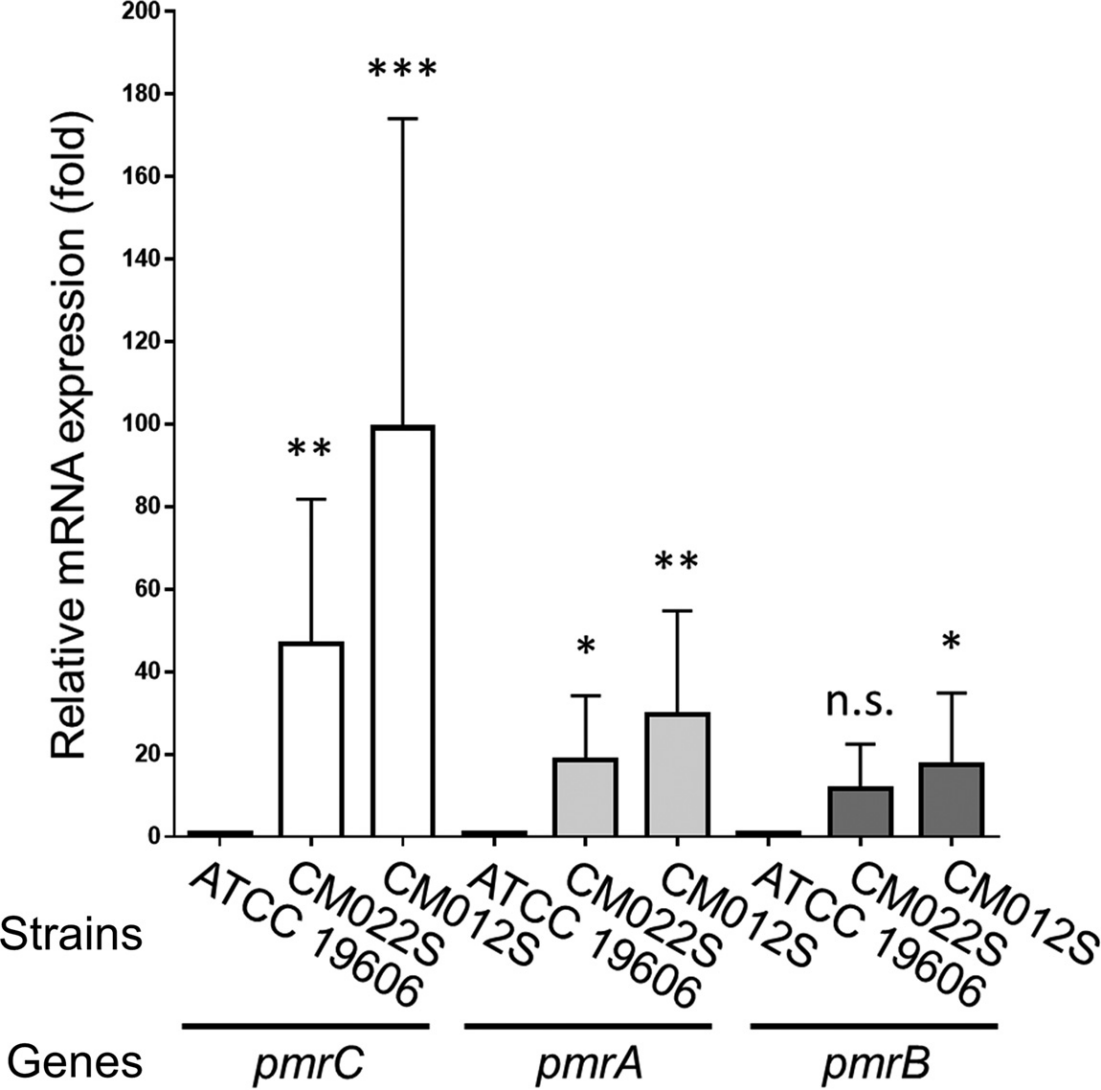
The expression of *pmrCAB* mRNA in representative A. baumannii strains of *pmrAB* mutants (CM022S [*pmrA*], CM012S [*pmrB*], and wild-type strain ATCC 19606) was analyzed by qRT-PCR. The strains were grown in LB broth at 37°C with shaking until they reached an OD_600_ of 0.75. Total RNA was extracted and subjected to qRT-PCR by the intercalator method (TB Green). Data are means and SD; *n *= 3 per group, compared by one-way ANOVA. n.s., not significant; ***, *P* < 0.001; **, *P* < 0.01; *, *P* < 0.05.

The quantity of LPS was analyzed using representative strains of each mutation. Mutations in the *lpxACD* genes (KL001S, KL037S, and KL055S) resulted in endotoxin levels below the detection limit with a possible LPS-deficient phenotype. In contrast, endotoxin levels in the two *pmrAB* mutant strains (CM022S and CM012S) were not significantly different from that in the wild-type strain (Table 1). Mutations in *pmrAB* have been reported to increase the expression of the upstream *pmrC* gene, encoding lipid A phosphoethanolamine transferase, and *pmrAB* itself (26, 42, 46). Therefore, the expression level of *pmrCAB* mRNA in the representative *pmrAB* mutant strains was measured by quantitative reverse transcription-PCR (qRT-PCR). Levels of *pmrCAB* mRNA were enhanced in the *pmrAB* mutants compared to that in the wild-type strain (Fig. 5).

Previous studies have reported that the LPS-deficient A. baumannii strains have a higher biological cost than the LPS-modified strains (36, 47, 48). We confirmed the biological cost of representative strains by analyzing their proliferative and biofilm-forming activities. As expected, *lpxACD* mutant strains (KL001S, KL037S, and KL055S) exhibited considerably decreased proliferative and biofilm-forming activities than did the wild-type strain. However, growth defects and suppressed biofilm-forming activity were not observed in the *pmrAB* mutant strains (CM022S and CM012S) (Fig. S2A and B).

In addition to antimicrobials, we analyzed the MICs of the disinfectants ethanol, H_2_O_2_, sodium dodecyl sulfate (SDS), and benzalkonium in the representative strains (Table 2). The MICs of disinfectants in the *pmrAB* mutant strains were almost the same as those in the wild-type strain. In contrast, the MICs of SDS, benzalkonium, and anionic and cationic detergents for the *lpxACD* mutant strains were decreased. The MICs of ethanol and H_2_O_2_ did not change in any strains. These results suggest that *pmrAB* mutant strains are likely to be more pathogenic than *lpxACD* mutant strains. It is also noteworthy that the MICs of some antimicrobials and disinfectants, such as tigecycline, ethanol, and H_2_O_2_, did not decrease in the *lpxACD* mutant strains despite complete loss of LPS.

## DISCUSSION

In this study, we focused on the mechanism by which A. baumannii acquire resistance to colistin under the selective pressure of colistin alone and in combination with other antimicrobials. Many patients with infections caused by colistin-resistant A. baumannii undergo colistin treatment before isolation of the colistin-resistant strain, and it is hypothesized that colistin resistance predominantly emerges under selective pressure during colistin therapy in individual patients (22, 49). There are reports that most clinically isolated colistin-resistant strains are LPS-modified strains (20, 23, 38, 50), which was also the case in our *in silico* analysis. Consistent with the findings of the present study, a recent systemic review (37) concluded that the *in vivo* emergence of colistin resistance in A. baumannii is mediated by mutations in *pmrAB* rather than by LPS loss. Although LPS-deficient strains were predominantly obtained when colistin was used alone for selection, we observed preferential selection of *pmrAB* mutants, probably harboring a modified LPS, when meropenem was added to the selection medium *in vitro*. These results are consistent with a previous report that when colistin and other antimicrobial agents were used to treat carbapenem-resistant A. baumannii in clinical settings, resistance developed during treatment, resulting in an LPS-modified colistin-resistant strain (22).

The underlying mechanism for the preferential selection of *pmrAB* mutants was further deciphered. As the spectrum of mutants obtained through selection is based on both frequency of occurrence and fitness of each mutant, it is necessary to analyze both factors. To further investigate this, we evaluated the bactericidal activity of various antimicrobial agents toward LPS-deficient (KL001S) and LPS-modified (CM012S) strains. We observed that the LPS-deficient strains could not proliferate in the presence of colistin and sub-MIC of meropenem, while no such defects were observed in the LPS-modified strains under the same conditions (Fig. S3). These results suggest that the main mechanism of preferential selection of *pmrAB* mutants may be that LPS-deficient strains are sensitive to colistin and sub-MIC of meropenem. However, the frequency of *pmrAB* mutants might be higher when colistin and meropenem are simultaneously added to the selection medium than when only colistin is added. The *pmrAB* mutants grew well even when meropenem was added to the medium, and the population plated onto the selection plates contained a similar number of *pmrAB* mutants, suggesting that *pmrAB* mutations occurred under selection pressure. We further sought to determine whether the addition of meropenem exerted mutagenic effects on A. baumannii. Therefore, the frequency of resistance to rifampicin, a commonly used drug during mutation rate analysis, was measured (51). Contrary to expectations, concomitant use of 1/5× MIC of meropenem and 5× MIC of rifampicin (10 μg/mL) did not alter the frequency of rifampicin-resistant colonies (Fig. S4). On the other hand, colistin resistance by LPS modification was dependent on the activity of phosphoethanolamine transferase, which was determined by both expression level and specific activity. This assumption prompted us to consider another possibility: that the addition of low-dose meropenem or ciprofloxacin enhanced the activity of phosphoethanolamine transferase, resulting in preferential selection of *pmrAB* mutants. The combination with antimicrobials might give rise to a complex selection mechanism, which is one limitation of the present study. Future research should focus on determining the appropriate antimicrobial agents to be used in combination with colistin to change the mutation spectrum and elucidate the detailed mechanism.

In this study, all colistin-resistant strains harbored mutations in *pmrAB* genes after concomitant use of meropenem. PmrAB is a two-component regulatory system that responds to presently unidentified signal(s), although it can also be activated by specific amino acid changes (42). Activation of the PmrAB system enhances the production of LPS-modifying enzymes. Most clinical isolates have mutations in PmrB, a membrane-bound histidine kinase. Among the frequently detected amino acid substitutions, P233S, R263C, and A226V were also identified in this study; however, P170L and P360Q were not (41, 42, 50). Most of the mutation sites detected in this study were located at specific sites in either the response regulatory domain of PmrA or the histidine kinase domain of PmrB. However, it has not been experimentally tested whether mutations in most clinical isolates are truly responsible for colistin resistance in A. baumannii. One study reported that a colistin-susceptible A. baumannii strain harbored the A226V substitution in PmrB, suggesting that PmrAB mutations alone do not confer colistin resistance (52). These findings suggest the importance of considering not only the single nucleotide polymorphisms in genes involved in colistin resistance but also their genetic background, as also pointed out by Gerson et al. (53). By confirming isogenic *pmrAB* mutants, we succeeded in producing mutation libraries in the background of two A. baumannii strains, ATCC 19606 and ATCC BAA-1605, and present useful data for understanding the colistin resistance mechanism and the PmrAB two-component system.

Insertion sequences (IS) are involved in colistin resistance in A. baumannii. In addition to gene disruption by IS insertion, transposition of IS*Aba1* containing an outward promoter upstream of *eptA*, a *pmrC* homolog, enhances the expression of phosphoethanolamine transferase, enabling LPS modification (53, 54). Transpositions of IS were observed in most colistin-resistant strains generated in this study; IS*Aba11* was observed in more than 30% of the colistin-resistant isolates of ATCC 19606 (Fig. 1A; Tables S1 and 2). Even with the ATCC BAA-1605 strain lacking IS*Aba11*, other IS were present in more than 40% of the colistin-resistant strains (Table S8). In agreement with our results, previous reports have shown that IS*Aba11* mediates LPS deficiency through insertional inactivation of *lpxACD* genes. Moreover, a high number of insertions in *lpxC* compared to those in *lpxA* and *lpxD*, a semiconserved AT-rich consensus sequence upstream of the IS*Aba11* insertion site, has been reported, suggesting that IS*Aba11* insertion sites may be sequence dependent (55, 56). In addition to these reports, we analyzed the ratio and frequency of IS*Aba11* insertions, substitutions, and short indels (Fig. 1B). Furthermore, Olmeda-Lopez et al. reported that antimicrobials affect IS insertion; the sub-MIC of tetracycline significantly increased IS*Aba11* insertion, and rifampicin completely inhibited the emergence of colistin resistance due to IS*Aba11* inactivation of *lpxC* gene (55). IS may be involved, because the mutational spectrum of colistin resistance was altered in this study with sub-MIC antimicrobials. The detailed relationship between IS and colistin resistance mechanisms should be explored in future studies.

**FIG 6:**
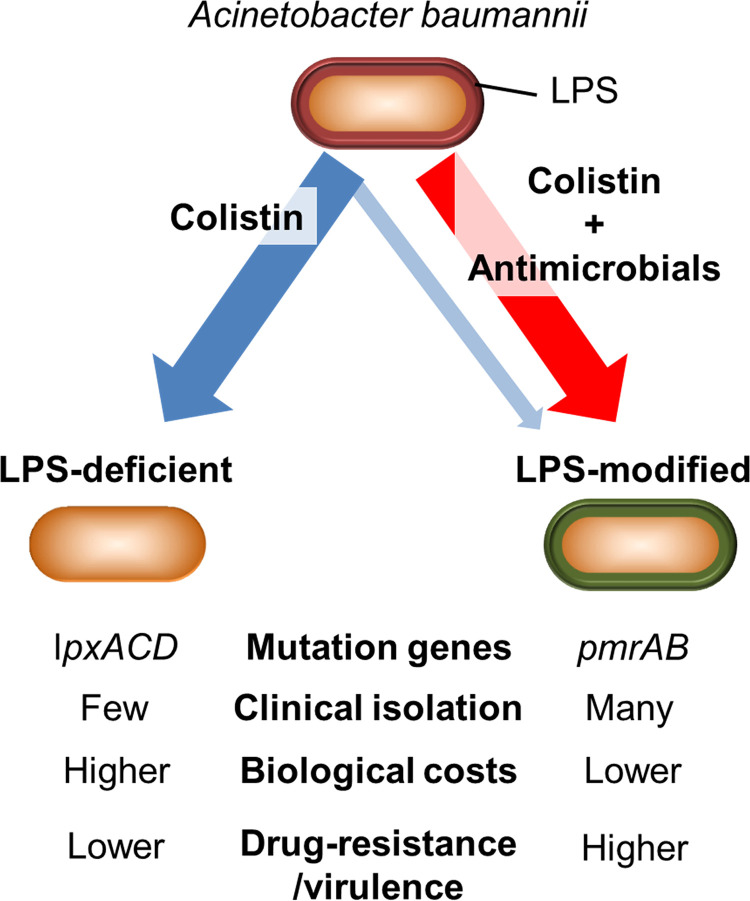
Proposed schematic diagram of the present study. A. baumannii was treated with colistin alone, which mainly selected mutations in *lpxACD* genes, resulting in LPS-deficient colistin-resistant strains. On the other hand, strains with modified LPS due to mutations in *pmrAB* were generated on treatment with combination of antimicrobials. LPS-modified colistin-resistant strains exhibit lower biological costs and higher drug resistance/virulence than LPS-deficient strains.

The schematic representation of the present study is shown in Fig. 6. LPS-deficient A. baumannii is more sensitive to host antimicrobials, such as lysozyme and lactoferrin (35, 57). We demonstrated that the LPS-modified strains had higher virulence than LPS-deficient strains in terms of antimicrobial and disinfectant resistance, proliferation, and biofilm-forming ability (Table 2 and Fig. S2), suggesting that colistin-resistant LPS-modified A. baumannii strains may be more difficult to treat than LPS-deficient strains in clinical settings. The LPS-modified strains selected using a combination of low-dose antimicrobials harbored mutations in *pmrAB* genes. These observations support the fact that most clinically isolated colistin-resistant strains are LPS-modified strains. Although the concomitant use of colistin and carbapenems has been reported to have a synergistic effect *in vitro* against carbapenem-resistant A. baumannii strains, and clinical trials have also been conducted to evaluate the same (58–63), our insights strongly suggest the importance of monitoring the emergence of LPS-modified strains during treatment. Additionally, the results of clinical trials suggest that combination therapy with colistin and meropenem is not superior to colistin monotherapy for carbapenem-resistant A. baumannii strains (64). Notably, a *post hoc* analysis of the AIDA study (the multicenter open-label randomized controlled trial study to compare colistin alone and combinational treatment of colistin and meropenem against severe infections caused by carbapenem-resistant Gram-negative infections) reported higher mortality for the cases where infection was treated with colistin-meropenem combination than with colistin alone in carbapenem-resistant A. baumannii infections (65). Hence, further investigation and reassessment of relevant methodology is needed to identify clinically useful synergistic combinations against A. baumannii (9, 58). The findings of this study suggest that substantial solutions and precautions should be taken for facilitating current research and clinical treatment of colistin-resistant A. baumannii infections.

## MATERIALS AND METHODS

### Bacterial strains, growth conditions, and isolation of colistin-resistant strains.

The type strain A. baumannii ATCC 19606 and ATCC BAA-1605 were purchased from ATCC (Manassas, VA, USA). Bacteria were cultured for 18 h in Luria-Bertani (LB) broth (BD Biosciences, San Diego, CA, USA) at 37°C with constant shaking (135 rpm). Colistin-resistant strains of A. baumannii were isolated by directly plating the parent strain onto LB agar (BD Biosciences) containing 10 μg/mL (5× MIC) of colistin sulfate (Fujifilm Wako Pure Chemical Industries, Osaka, Japan) for 24 h at 37°C (29). LPS-deficient strains were cultured for 24 h in LB broth at 37°C with constant shaking (180 rpm).

### PCR-Sanger sequencing.

Genomic regions containing *lpxACD* and *pmrAB* genes were amplified by colony PCR using following specific primers (primer sequences are listed in Table 3): for *lpxAD*, lpxA_F and lpxD_R; for *lpxC*, lpxC_F1 and lpxC_R1 or lpxC_F2 and lpxC_R2; and for *pmrAB*, pmrA_F1 and pmrB_R. The PCR products were purified using the FastGene gel/PCR extraction kit (Nippon Genetics, Tokyo, Japan). Direct sequencing of each gene was performed by Eurofins Genomics (Tokyo, Japan) using the following primers: for *lpxA* from ATCC 19606, lpxA_F and lpxA_R1; for *lpxC*, lpxC_F1 and lpxC_R1 or lpxC_F2 and lpxC_R2; for *lpxD*, lpxD_F and lpxD_R; for *pmrAB*, pmrA_F1, pmrA_F2, and pmrB_R; and for *lpxA* from ATCC BAA-1605, lpxA_F and lpxA_R2.

**TABLE 3:** Primers

Primer	Sequence
lpxA_F	5′-TGGTAATGCAGAAGCGCGGTATCTACAA-3′
lpxA_R1	5′-ATCCTCTAGAGTCGACCAATATTCAAAGTCTGAAGAAGCA-3′
lpxA_R2	5′-CTGTGTCAGCAAATCAATACAAG-3′
lpxC_F1	5′-TCAGCAACGTAAGTAATTTAGCGTACA-3′
lpxC_R1	5′-GCCAAGCTTTACTACGTTTGGCAAGCAA-3′
lpxC_F2	5′-GCAGAGCCAAGAAAGCGTAA-3′
lpxC_R2	5′-AAATGTTACGTAGTGCCGCC-3′
lpxD_F	5′-AAGCTTGCATGCGTTAAGCAAGCTGCTGAGCAATTACGAA-3′
lpxD_R	5′-CCAATAAGAATGGGTAACGATGCGGCAA-3′
pmrA_F1	5′-CCAACAAACTAAACAAAAGTTAA-3′
pmrA_F2	5′-TTGAACAGCATATTGCGACGTT-3′
pmrB_R	5′-GCAAATGATGCGAGGAGCACAT-3′
qPCR_pmrC_F	5′-TTCTCGGGTATGCCACGTGTA-3′
qPCR_pmrC_R	5′-CCGCACGTTTTGCAATATCTAGT-3′
qPCR_pmrA_F	5′-TGATGAGTTGCTTGCCCGTAT-3′
qPCR_pmrA_R	5′-ATAGTTGATCTTGACTCGCAAGTTGA-3′
qPCR_pmrB_F	5′-CGTTTTTATCGCGTGCATCA-3′
qPCR_pmrB_R	5′-AAGCCTTTGAGTTGCACGATCT-3′
qPCR_16S rRNA_F	5′-CATGAAGTCGGAATCGCTAGTAATC-3′
qPCR_16S rRNA_R	5′-TGACGGGCGGTGTGTACA-3′
qPCR_lpxC_F	5′-TTGTGGAAGTGTCTGCTTCTGAAG-3′
qPCR_lpxC_R	5′-GCCACCTTGCATGAGCAAAT-3′
qPCR_lpxD_F	5′-CGGGAACCGGATTATTTGAAA-3′
qPCR_lpxD_R	5′-GGTCAATGGCACATCTGCTAATT-3′

### Analysis of NCBI BioSample isolates.

We analyzed LpxACD and PmrAB mutations in colistin-resistant A. baumannii strains from clinical isolates available in the NCBI BioSample database with published whole genomes. Briefly, we compared the LpxACD and PmrAB amino acid sequences of 265 colistin-susceptible A. baumannii strains, defined the mutations found in more than 1% of the strains as single nucleotide polymorphisms, and created reference sequences. Mutation analysis of strains determined to be colistin resistant with a MIC of ≥4 μg/mL was performed using the above-mentioned sequences as queries. The results of this analysis are presented in Table S4.

### Frequency analysis of the emergence of drug-resistant strains and isolating methods.

Bacterial culture density was adjusted with LB broth to an optical density at 600 nm (OD_600_) of 1.0 using overnight cultures. The culture OD_600_ was measured using a CO8000 Biowave (WPA; Biochrom, Cambridge, UK). The frequency of colistin resistance was analyzed by culturing 0.2 mL (for ATCC 19606) or 0.1 mL (for ATCC BAA-1605) of bacterial suspension (OD_600_ of 1.0) for 24 h on LB agar plates containing 10 μg/mL (5× MIC) of colistin at 37°C. The frequency of colistin resistance with a combination of colistin and 1/5× MIC of meropenem (Fujifilm Wako Pure Chemical Industries) was measured using 0.1 mL of bacterial culture (OD_600_ of 10 for ATCC 19606 or 25 for ATCC BAA-1605). Bacterial suspensions were plated on LB agar containing 10 μg/mL of colistin and 0.1 μg/mL (for ATCC 19606) or 2 μg/mL (for ATCC BAA-1605) of meropenem. To calculate the number of CFU, 0.1 mL of OD_600_ 1.0 culture diluted to 10^−7^ (for ATCC 19606) or 10^−8^ (for ATCC BAA-1605) was plated on nonantibiotic LB agar. Also, overlapping colonies were counted when the frequency was calculated. The frequency of colistin resistance in the presence of colistin and 1/5× MIC (0.4 μg/mL) of ciprofloxacin (Fujifilm Wako Pure Chemical Industries) was measured by plating 0.1 mL of OD_600_ 10 culture for ATCC 19606, as described above.

The frequency of resistance to rifampicin alone or in combination with 1/5× MIC of meropenem was measured using 0.1 mL of overnight culture (OD_600_ of approximately 30); the culture was plated on LB agar plates containing 10 μg/mL (5× MIC) of rifampicin alone or in combination with 0.1 μg/mL meropenem.

### Whole-genome sequencing.

Genomic DNA was isolated from overnight cultures using a DNeasy blood and tissue kit (Qiagen, Hilden, Germany). Sequencing libraries were prepared using a NEBNext Ultra II FS DNA library preparation kit for Illumina (New England BioLabs, Ipswich, MA, USA) according to the manufacturer’s instructions. The libraries were subjected to 2 × 150-bp sequencing in an Illumina HiSeq X sequencer (Illumina, San Diego, CA, USA). To obtain the complete reference genome sequence of the parental strain, we conducted a long-read sequencing analysis using Oxford Nanopore F sequencing technology (Oxford Nanopore, Oxford, UK). After the generation of the barcoded library using a ligation sequencing kit (Oxford Nanopore) with native barcoding expansion (Oxford Nanopore), the library was subjected to sequence analysis using the MinION Mk1C (Oxford Nanopore) and sequencing kit (Oxford Nanopore). The circular genome sequence was assembled using Unicycler software (66). Open reading frames (ORFs), and RNA regions were annotated by Prokka v1.14.6 (67) using GCF_000737145.1_ASM73714v1 as the primary database. Some ORFs of interest were manually annotated.

### Determination of antibiotic and disinfectant MICs.

MICs of antibiotics and disinfectants were determined by microdilution in LB broth for antibiotics or Mueller-Hinton broth (BD Biosciences) for disinfectants, as described previously (35). Antibiotic agents, such as colistin, meropenem, amikacin, and ciprofloxacin, and disinfectants, such as ethanol, H_2_O_2_, SDS, and benzalkonium, were purchased from Fujifilm Wako Pure Chemical Industries. Tigecycline was purchased from the Tokyo Chemical Industry (Tokyo, Japan). The bacterial culture was adjusted to an OD_600_ of 0.001 from the overnight cultures. MICs were monitored using a 2-fold dilution series for each antibiotic or disinfectant.

### LAL assay.

The *Limulus* amebocyte lysate (LAL) assay was performed using a Toxicolor LS-50M set (Seikagaku Corporation, Tokyo, Japan) according to the manufacturer’s protocol. The bacterial culture was adjusted to an OD_600_ of 0.1 from overnight cultures, and the cultures were diluted in sterile, pyrogen-free saline (Otsuka Pharmaceutical Co., Ltd., Tokyo, Japan).

### Quantitative qRT-PCR.

Cultures of A. baumannii were grown in LB broth at 37°C with constant shaking until the OD_600_ reached 0.75. The cells were harvested from 1 mL culture by centrifugation, and total RNA was prepared using the acid guanidinium thiocyanate-phenol-chloroform extraction (AGPC) method using TRIzol (Thermo Fisher Scientific, Waltham, MA, USA) according to the manufacturer’s protocol. DNA contamination was removed by treatment with DNase I (Nippon Gene, Tokyo, Japan), according to the manufacturer’s instructions. RNA concentration was quantified using NanoDrop One (Thermo Fisher Scientific). Total RNA (500 ng) was reverse transcribed using PrimeScript RT master mix (TaKaRa Bio Inc., Shiga, Japan) according to the manufacturer’s protocol. TB Green Premix Ex Taq II (Tli RNaseH Plus) was purchased from TaKaRa Bio, Inc., and the Applied Biosystems StepOne Plus real-time PCR system (Thermo Fisher Scientific) was used to perform RT-qPCR. Table 3 lists the primers used for PCR. Data were generated using cDNA prepared from three independent RNA isolations, and qRT-PCR was performed in triplicate to ensure accuracy. Fold changes in gene expression relative to the control strain (ATCC 19606) and the control gene (16S rRNA gene) were determined using the 2^−ΔΔ^*^CT^* method (68).

### Growth curve.

Bacterial culture density was adjusted to an OD_600_ of 0.001 using overnight cultures. Bacteria were grown under static conditions in LB broth for 24 h at 37°C and the absorbance at 595 nm (*A*_595_ nm) was measured every hour using Multiskan FC (Thermo Fisher Scientific).

### Biofilm formation assay.

The biofilm-forming ability of each strain was analyzed using the crystal violet staining method. Bacterial culture density was adjusted to an OD_600_ of 0.01 using overnight cultures. Bacteria were grown under static conditions for 24 h in LB broth at 37°C in a 96-well U-bottom plate (163320, Thermo Fisher Scientific). Following incubation, the culture was carefully removed, the plates were washed three times with phosphate-buffered saline (PBS), and each well was stained for 15 min with 0.1% crystal violet at about 25°C. After gently removing the crystal violet solution, each well was washed three times with PBS to remove excess stain and dried for 2 h at 37°C. The remaining crystal violet was solubilized in 95% (vol/vol) ethanol by incubating for 5 min. The eluate was transferred to a new plate, and *A*_570_ was measured (Varioskan; Thermo Fisher Scientific). Fold changes in biofilm formation were calculated using the control strain (ATCC 19606) at A_570_ of 1.0.

### Bactericidal activity of colistin alone and in combination with meropenem.

Bacterial culture density was adjusted with LB broth to an OD_600_ of 1.0 using overnight cultures. The culture was serially diluted (10^−1^ to 10^−9^), and 10 μL of each dilution was spotted on LB agar plates containing 10 μg/mL (5× MIC) of colistin alone or in combination with 0.1 μg/mL (1/5× MIC) of meropenem or no antibiotic. The CFU on non-antibiotic-containing LB agar was used to calculate the percent surviving bacteria. In this experiment, A. baumannii KL001S and CM012S were used as the LPS-deficient and LPS-modified strains, respectively, and ATCC 19606 was the wild-type strain.

### Statistical analysis.

Statistical analysis was performed using GraphPad Prism software (GraphPad Software, San Diego, CA, USA). Data are expressed as means and standard deviations (SD) and were compared using the Mann-Whitney *U* test and one-way and two-way analysis of variance (ANOVA). Differences with *P* values of <0.05 were considered statistically significant.

### Data availability.

Original data sets are available in a publicly accessible repository. The whole-genome sequencing data of the representative A. baumannii isolates in this study were registered with the DDBJ (BioProject accession no. PRJDB12922). The GenBank file for the complete reference genome sequence of ATCC 19606 was registered with the DDBJ (nucleotide accession no. AP025740).
