# Supplementary material for: Preferential Selection of Low-Frequency, Lipopolysaccharide-Modified, Colistin-Resistant Mutants with a Combination of Antimicrobials in Acinetobacter baumannii
Source: Microbiol Spectr. 2022 Sep 29;10(5):e01928-22. doi: 10.1128/spectrum.01928-22 (PMC9602988; doi:10.1128/spectrum.01928-22)
Supplement: Supplemental file 1 — Supplemental material. Download spectrum.01928-22-s0001.pdf, PDF file, 0.6 MB [file spectrum.01928-22-s0001.pdf]

Supplemental
Table 1
Kamoshida et al.

Colistin

	lpxA	*lpxC*	*lpxD*	*pmrA*	*pmrB*	**Total**
**in/del**	4 (3)	13 (11)	1 (1)	0	0	18 (15)
**Substitution**	13 (6)	12 (5)	4 (2)	0	0	29 (13)
**IS*Aba11***	4 (2)	11 (7)	9 (6)	0	0	24 (15)
**Total**	21 (11)	36 (22)	14 (9)	0	0	71 (43)

Colistin+Meropenem

	*lpxA**	*lpxC**	*lpxD**	*pmrA*	*pmrB*	**Total**
**in/del**	0	0	0	0	1	0
**Substitution**	0	0	0	12 (4)	28 (19)	41 (23)
**IS*Aba11***	0	0	0	0	0	0
**Total**	0	0	0	12 (4)	29 (19)	41 (23)

The numerical value is the number of isolated strains, and (numbers) are the number of independent events.
* 21 of 41 strains were analyzed.

Supplemental
Table 2
Kamoshida et al.

ATCC 19606 Colistin-resistant strains

Name	Colony	Genes					Amino acid change	Mutation gene
		lpxA	*lpxC*	*lpxD*	*pmrA*	*pmrB*		
KL001	1	591_592delTA	-	-	-	-	I198fs(I198*)	*lpxA*
KL002	1	591_592delTA	-	-	-	-	I198fs(I198*)	*lpxA*
KL003	1	-	-	-22bp>IS*Aba11* (AAG^GTT)	-	-		*lpxD*
KL004	2	-	394>IS*Aba11* (Inversion)	-	-	-	L132fs(L132*)	*lpxC*
KL005	3	82delT	-	-	-	-	I28fs(I28Y)V35*	*lpxA*
KL006	3	-	-	-	-	-		Not detected
KL007	3	-	-	-	-	-		Not detected
KL008	3	-	630delT	-	-	-	L211fs(L211*)	*lpxC*
KL009	3	461G>A	-	-	-	-	G154D	*lpxA*
KL010	3	562G>A	-	-	-	-	G188S	*lpxA*
KL011	3	-	-	-	-	-		Not detected
KL012	3	562G>A	-	-	-	-	G188S	*lpxA*
KL013	3	562G>A	-	-	-	-	G188S	*lpxA*
KL014	3	562G>A	-	-	-	-	G188S	*lpxA*
KL015	3	562G>A	-	-	-	-	G188S	*lpxA*
KL016	3	461G>A	-	-	-	-	G188S	*lpxA*
KL017	3	562G>A	-	-	-	-	G188S	*lpxA*
KL018	3	562G>A	-	-	-	-	G188S	*lpxA*
KL019	4	-	119G>C	-	-	-	R40P	*lpxC*
KL020	5	-	-	-	-	-		Not detected
KL021	5	-	327_330insATTT	-	-	-	L114fs(L114F)Q122*	*lpxC*
KL022	5	315G>A	-	-	-	-	M117I	*lpxA*
KL023	5	-	563C>T	-	-	-	A188V	*lpxC*
KL024	5	-	394>IS*Aba11* (Inversion)	-	-	-	L132*	*lpxC*
KL025	5	-	563C>T	-	-	-	A188V	*lpxC*
KL026	5	-	563C>T	-	-	-	A188V	*lpxC*
KL027	5	-	563C>T	-	-	-	A188V	*lpxC*
KL028	5	-	563C>T	-	-	-	A188V	*lpxC*
KL029	5	-	563C>T	-	-	-	A188V	*lpxC*
KL030	6	-	97_98delGT	-	-	-	V33fs(V33G)A52*	*lpxC*
KL031	7	-	569C>T	-	-	-	T190I	*lpxC*
KL032	7	-	-	233>IS*Aba11*	-	-	I78fs(I78G)P83*	*lpxD*
KL033	8	-	391>IS*Aba11* (Inversion)	-	-	-	I131fs(I131*)	*lpxC*
KL034	8	-	391>IS*Aba11* (Inversion)	-	-	-	I131fs(I131*)	*lpxC*
KL035	8	-	391>IS*Aba11* (Inversion)	-	-	-	I131fs(I131*)	*lpxC*
KL036	8	-	391>IS*Aba11* (Inversion)	-	-	-	I131fs(I131*)	*lpxC*
KL037	9	-	475_689(215bp)del	-	-	-	D159fs(D159C)D161*	*lpxC*
KL038	9	469G>A	-	-	-	-	G157R	*lpxA*
KL039	9	-	-	-	-	-		Not detected
KL040	9	-	429delA	-	-	-	A144fs(A144Q)M194*	*lpxC*
KL041	9	-	-	749C>T	-	-	T250I	*lpxD*
KL042	10	-	327_330delATTT	-	-	-	Y112fs(Y112C)F128*	*lpxC*
KL043	10	263>IS*Aba11* (Inversion)	-	-	-	-	N88fs(N88I)S96*	*lpxA*
KL044	10	-	327_330delATTT	-	-	-	Y112fs(Y112C)F128*	*lpxC*
KL045	10	-	-	-3bp>IS*Aba11* (Inversion)(AAa^tta)	-	-		*lpxD*
KL046	10	-	327_330delATTT	-	-	-	Y112fs(Y112C)F128*	*lpxC*
KL047	11	-	327_330delATTT	-	-	-	Y112fs(Y112C)F128*	*lpxC*
KL048	11	413>IS*Aba11*	-	-	-	-	V140fs(V140L)H143*	*lpxA*
KL049	11	413>IS*Aba11*	-	-	-	-	V140fs(V140L)H143*	*lpxA*
KL050	11	-	394>IS*Aba11* (Inversion)	-	-	-	L132fs(L132*)	*lpxC*
KL051	11	413>IS*Aba11*	-	-	-	-	V140fs(V140L)H143*	*lpxA*
KL052	11	-	-	271A>C	-	-	T91P	*lpxD*
KL053	11	-	-	271A>C	-	-	T91P	*lpxD*
KL054	11	-	-	271A>C	-	-	T91P	*lpxD*
KL055	11	-	-	218insA	-	-	D75fs(D75R)N76*	*lpxD*
KL056	12	-	-	-	-	-		Not detected
KL057	12	-	-	-	-	-		Not detected
KL058	12	306_310delGCAA	-	-	-	-	Q103fs(Q103I)A106*	*lpxA*
KL059	12	-	-	232>IS*Aba11* (Inversion)	-	-	I78fs(I78*)	*lpxD*
KL060	12	-	-	232>IS*Aba11* (Inversion)	-	-	I78fs(I78*)	*lpxD*
KL061	13	-	327_330insATTT	-	-	-	Y112fs(Y112C)F128*	*lpxC*
KL062	13	-	392>IS*Aba11*	-	-	-	L132fs(L132G)E136*	*lpxC*
KL063	13	-	703G>T	-	-	-	V235F	*lpxC*
KL064	13	-	703G>T	-	-	-	V235F	*lpxC*
KL065	13	-	811>IS*Aba11*	-	-	-	L271fs(L271*)	*lpxC*
KL066	13	-	703G>T	-	-	-	V235F	*lpxC*
KL067	13	-	-	-	-	-		Not detected
KL068	13	-	-35bpA>G	-	-	-		*lpxC*
KL069	13	-	811>IS*Aba11*	-	-	-	L271fs(L271*)	*lpxC*
KL070	13	-	-	-2bp>IS*Aba11* (Inversion)(Aat^taA)	-	-		*lpxD*
KL071	14	-	327_330insATTT	-	-	-	L114fs(L114F)Q122*	*lpxC*
KL072	14	-	327_330delATTT	-	-	-	Y112fs(Y112C)F128*	*lpxC*
KL073	15	-	327_330delATTT	-	-	-	Y112fs(Y112C)F128*	*lpxC*
KL074	15	-	-	-	-	-		Not detected
KL075	15	-	-	-	-	-		Not detected
KL076	15	604C>T, 606A>T	-	-	-	-	R202C	*lpxA*
KL077	15	-	-	-	-	-		Not detected
KL078	15	-	-	-	-	-		Not detected
KL079	15	-	-	-	-	-		Not detected
KL080	15	-	-	-	-	-		Not detected
KL081	15	604C>T	-	-	-	-	R202*	*lpxA*
KL082	15	-	394>IS*Aba11*	-	-	-	L132fs(L132*)	*lpxC*
KL083	16	-	-	-22bp>ISAba11(AAG^GTT)	-	-		*lpxD*
KL084	16	-	-	-22bp>ISAba11(AAG^GTT)	-	-		*lpxD*
KL085	16	-	-	-22bp>ISAba11(AAG^GTT)	-	-		*lpxD*

Supplemental
Table 3
Kamoshida et al.

Mutation gene	Name	Mutation	Colistin MIC (μg/ml)
lpxA	KL001	591_592delTA: I198fs	128
lpxA	KL009	461G>A: G154D	128
lpxA	KL010	562G>A: G188S	128
lpxA	KL022	315G>A: M117I	256
lpxA	KL038	469G>A: G157R	128
lpxA	KL076	604C>T, 606A>T: R202C	128
lpxC	KL037	475_689(215)del: D159fs	64
lpxC	KL019	119G>C: R40P	128
lpxC	KL023	563C>T: A188V	256
lpxC	KL031	569C>T: T190I	128
lpxC	KL063	703G>T: V235F	256
lpxD	KL055	218insA: D75fs	64
lpxD	KL041	749C>T: T250I	128
lpxD	KL052	271A>C: T91P	8

Supplemental
Table 4
Kamoshida et al.

NCBI BioSample

BioSample ID	Amino acids					Colistin MIC	Mutation
	LpxA	LpxC	LpxD	PmrA	PmrB		
12087735	-	-	-	-	A138T, R263H	>256	PmrB
4901700	-	-	-	K172I	-	8	PmrA
4901699	-	-	-	K172I	-	8	PmrA
4901698	-	-	-	-	P170S	8	PmrB
4901697	-	-	-	-	P170L	>8	PmrB
4901694	-	-	-	-	-	4	Not detected
4901693	-	-	-	-	H266Y, Q270P	>8	PmrB
4901678	-	-	-	-	T235I	>8	PmrB
6837835	-	-	-	D10E	V133G, P154T	>4	PmrAB
6837830	-	-	-	-	L33P	>4	PmrB
6837819	-	-	-	-	20bp>IS*Aba125* (insersion)	>4	PmrB
6837812	-	-	-	-	-	>4	Not detected
6837767	-	-	-	-	L239I	>4	PmrB
6837762	-	-	-	-	-	>4	Not detected
6837757	-	-	-	-	-	>4	Not detected
6837753	-	-	D75G	-	-	4	LpxD
6837751	-	-	-	M6K	-	>4	PmrB
6827875	-	-	-	I173F	-	>4	PmrB
6892294	-	-	S100T	-	-	>4	LpxD
6892290	-	-	-	-	P170L, N337H	>4	PmrB
6892289	-	-	-	I173F	-	>4	PmrA
4254730	-	-	-	E8D	Y116H	>256	PmrAB
4254728	-	-	-	-	S17R	192	PmrB
4254727	-	-	-	-	T232I	128	PmrB

Supplemental
Table 5
Kamoshida et al.

ATCC 19606 Colistin-meropenem-resistant strains

Name	Colony	Genes					Amino acid change	Mutation gene
		lpxA	*lpxC*	*lpxD*	*pmrA*	*pmrB*		
CM001	1	-	-	-	-	695C>T	T232I	*pmrB*
CM002	1	-	-	-	-	695C>T	T232I	*pmrB*
CM003	2	-	-	-	-	692G>T	R231L	*pmrB*
CM004	2	-	-	-	-	692G>T	R231L	*pmrB*
CM005	2	-	-	-	-	692G>T	R231L	*pmrB*
CM006	2	-	-	-	-	692G>T	R231L	*pmrB*
CM008	2	-	-	-	-	692G>T	R231L	*pmrB*
CM011	3	-	-	-	-	680C>T	A227V	*pmrB*
CM012	3	-	-	-	-	697C>T	P233S	*pmrB*
CM015	3	-	-	-	-	680C>T	A227V	*pmrB*
CM016	3	-	-	-	-	680C>T	A227V	*pmrB*
CM017	3	-	-	-	-	680C>T	A227V	*pmrB*
CM018	4	-	-	-	-	787C>G	R263G	*pmrB*
CM019	5	-	-	-	-	794A>C	Q265P	*pmrB*
CM020	6	-	-	-	-	697C>T	P233S	*pmrB*
CM021	7	-	-	-	-	787C>T	R263C	*pmrB*
CM022	7	-	-	-	36G>T	-	M12I	*pmrA*
CM023	7	-	-	-	36G>T	-	M12I	*pmrA*
CM024	8	-	-	-	-	42(AATTTTCAGTGTCATCTTAGGTTGTATTTT)71 duplication	28-37 position insertion +VILGCILIFS	*pmrB*
CM025	9	-	-	-	-	937G>T	D313Y	*pmrB*
CM026	9	-	-	-	-	937G>C	D313H	*pmrB*
CM027	10	N.T.	N.T.	N.T.	-	794A>C	Q265P	*pmrB*
CM028	11	N.T.	N.T.	N.T.	36G>T	-	M12I	*pmrA*
CM029	11	N.T.	N.T.	N.T.	-	680C>T	A227V	*pmrB*
CM031	12	N.T.	N.T.	N.T.	-	944G>A	G315D	*pmrB*
CM032	12	N.T.	N.T.	N.T.	-	944G>A	G315D	*pmrB*
CM033	12	N.T.	N.T.	N.T.	-	680C>T	A227V	*pmrB*
CM034	12	N.T.	N.T.	N.T.	-	704C>T	T235I	*pmrB*
CM035	13	N.T.	N.T.	N.T.	-	788G>A	R263H	*pmrB*
CM036	13	N.T.	N.T.	N.T.	-	788G>A	R263H	*pmrB*
CM038	14	N.T.	N.T.	N.T.	-	697C>T	P233S	*pmrB*
CM041	15	N.T.	N.T.	N.T.	34A>G	-	M12V	*pmrA*
CM042	15	N.T.	N.T.	N.T.	34A>G	-	M12V	*pmrA*
CM043	15	N.T.	N.T.	N.T.	34A>G	-	M12V	*pmrA*
CM044	15	N.T.	N.T.	N.T.	34A>G	-	M12V	*pmrA*
CM045	15	N.T.	N.T.	N.T.	34A>G	-	M12V	*pmrA*
CM046	15	N.T.	N.T.	N.T.	34A>G	-	M12V	*pmrA*
CM047	15	N.T.	N.T.	N.T.	-	787C>G	R263G	*pmrB*
CM048	15	N.T.	N.T.	N.T.	34A>G	-	M12V	*pmrA*
CM049	15	N.T.	N.T.	N.T.	34A>G	-	M12V	*pmrA*
CM051	16	N.T.	N.T.	N.T.	38G>T	-	I13S	*pmrA*

N.T. Not Tested

Supplemental
Table 6
Kamoshida et al.

ATCC 19606 Colistin-ciprofloxacin-resistant strains

Name	Colony			Amino acid change	Mutation gene
		pmrA	***pmrB***		
CpCL001	1	-	689C>T	A226V	*pmrB*
CpCL002	1	-	787C>T	R263C	*pmrB*
CpCL003	1	-	787C>T	R263C	*pmrB*
CpCL004	1	-	787C>T	R263C	*pmrB*
CpCL005	2	239C>T	-	A80V	*pmrA*
CpCL006	2	-	821T>G	L274W	*pmrB*
CpCL007	3	-	794A>C	Q265P	*pmrB*
CpCL008	3	-	944G>C	G315D	*pmrB*
CpCL009	4	-	697C>T	P233S	*pmrB*
CpCL010	4	-	49A>C	S17R	*pmrB*
CpCL011	5	-	676G>A	A226T	*pmrB*
CpCL012	5	-	676G>A	A226T	*pmrB*
CpCL013	6	-	680C>T	A227V	*pmrB*
CpCL014	6	-	692G>A	R231Q	*pmrB*
CpCL015	6	-	716T>C	L239S	*pmrB*
CpCL016	6	-	716T>C	L239S	*pmrB*
CpCL017	6	-	716T>C	L239S	*pmrB*

Supplemental
Table 7
Kamoshida et al.

ATCC BAA-1605 Colistin-resistant strains

Name	Colony	Genes		Amino acid change	Mutation gene
		pmrA	*pmrB*		
BAAC001	1	60G>C	-	L20F	*pmrA*
BAAC002	1	60G>T	-	L20F	*pmrA*
BAAC003	1	60G>T	-	L20F	*pmrA*
BAAC009	2	-	704C>T	T235I	*pmrB*
BAAC010	2	-	704C>T	T235I	*pmrB*
BAAC033	6	-	707C>A	T235I	*pmrB*
BAAC044	7	-	697C>T	P233S	*pmrB*
BAAC045	7	-	686A>C	E229A	*pmrB*
BAAC046	7	-	944G>T	G315V	*pmrB*
BAAC066	10	-	944G>A	G315D	*pmrB*
BAAC067	10	-	944G>A	G315D	*pmrB*

ATCC BAA-1605 Colistin-meropenem-resistant strains

Name	Colony	Genes		Amino acid change	Mutation gene
		pmrA	*pmrB*		
BAACM001	1	60G>C	-	L20F	*pmrA*
BAACM002	1	60G>T	-	L20F	*pmrA*
BAACM003	1	60G>T	-	L20F	*pmrA*
BAACM004	1	60G>C	-	L20F	*pmrA*
BAACM005	1	60G>T	-	L20F	*pmrA*
BAACM006	1	60G>T	-	L20F	*pmrA*
BAACM007	1	-	704C>T	T235I	*pmrB*
BAACM008	1	60G>C	-	L20F	*pmrA*
BAACM009	1	60G>C	-	L20F	*pmrA*
BAACM010	1	60G>T	-	L20F	*pmrA*
BAACM011	1	60G>T	-	L20F	*pmrA*
BAACM012	4	-	697C>T	P233S	*pmrB*
BAACM013	5	-	938A>T	D313V	*pmrB*
BAACM014	5	60G>C	-	L20F	*pmrA*
BAACM015	6	60G>C	-	L20F	*pmrA*
BAACM016	6	60G>C	-	L20F	*pmrA*
BAACM017	7	-	944G>T	G315V	*pmrB*
BAACM018	7	-	787C>G	R263G	*pmrB*
BAACM019	7	60G>C	-	L20F	*pmrA*
BAACM020	7	60G>C	-	L20F	*pmrA*
BAACM021	7	-	699A>C	E229D	*pmrB*
BAACM022	7	-	699A>C	E229D	*pmrB*
BAACM023	8	-	698A>C	P233H	*pmrB*
BAACM024	8	-	788G>A	R263H	*pmrB*
BAACM025	8	-	698A>C	P233H	*pmrB*
BAACM026	9	-	787C>G	R263G	*pmrB*
BAACM027	10	-	788G>A	R263H	*pmrB*

Supplemental
Table 8
Kamoshida et al.

IS insertion in ATCC BAA-1605 Colistin-resistant strains

Name	Colony	Genes			Remark
		lpxA	*lpxC*	*lpxD*	
BAAC001	1	-	-	-	*pmrA* mutation
BAAC002	1	-	-	-	*pmrA* mutation
BAAC003	1	-	-	-	*pmrA* mutation
BAAC004	1	-	-	○	-
BAAC005	1	-	○	-	-
BAAC006	1	-	○	-	-
BAAC007	1	-	○	-	-
BAAC008	1	-	-	-	*pmrB* mutation
BAAC009	2	-	-	-	*pmrB* mutation
BAAC010	2	-	-	-	-
BAAC011	2	-	-	-	-
BAAC012	2	-	○	-	-
BAAC013	2	-	○	-	-
BAAC014	2	-	-	-	-
BAAC015	2	○	-	-	-
BAAC016	2	-	○	-	-
BAAC017	3	-	-	-	-
BAAC018	3	-	-	-	-
BAAC019	3	-	○	-	-
BAAC020	3	-	○	-	-
BAAC021	4	-	○	-	-
BAAC022	4	-	○	-	-
BAAC023	4	-	○	-	-
BAAC024	4	-	-	-	-
BAAC025	5	-	○	-	-
BAAC026	5		-	-	-
BAAC027	5	-	-	-	-
BAAC028	5	-	○	-	-
BAAC029	5	-	-	-	-
BAAC030	5	-	-	-	-
BAAC031	5	-	○	-	-
BAAC032	5	-	-	-	-
BAAC033	6	-	-	-	*pmrB* mutation
BAAC034	6	-	○	-	-
BAAC035	6	-	-	-	-
BAAC036	6	-	○	-	-
BAAC037	6	-	○	-	-
BAAC038	6	-	-	-	-
BAAC039	6	-	○	-	-
BAAC040	6	-	-	-	-
BAAC041	6	-	-	-	-
BAAC042	6	-	-	-	-
BAAC043	6	-	○	-	-
BAAC044	7	-	-	-	*pmrB* mutation
BAAC045	7	-	-	-	*pmrB* mutation
BAAC046	7	-	-	-	*pmrB* mutation
BAAC047	7	-	-	-	-
BAAC048	7	-	-	-	-
BAAC049	7	-	-	-	-
BAAC050	7	-	-	-	-
BAAC051	7	-	-	-	-
BAAC052	7	-	-	-	-
BAAC053	7	-	-	-	-
BAAC054	7	-	○	-	-
BAAC055	7	-	○	-	-
BAAC056	7	-	-	○	-
BAAC057	7	○	-	-	-
BAAC058	8	-	-	-	-
BAAC059	8	-	○	-	-
BAAC060	9	-	○	-	-
BAAC061	9	-	-	○	-
BAAC062	9	-	○	-	-
BAAC063	9	-	-	-	-
BAAC064	9	-	-	-	-
BAAC065	9	-	-	-	-
BAAC066	10	-	-	-	*pmrB* mutation
BAAC067	10	-	-	-	*pmrB* mutation
BAAC068	10	-	○	-	-
BAAC069	10	-	○	-	-
BAAC070	10	-	○	-	-
BAAC071	10	-	○	-	-

Supplemental
Fig. 1
Kamoshida et al.

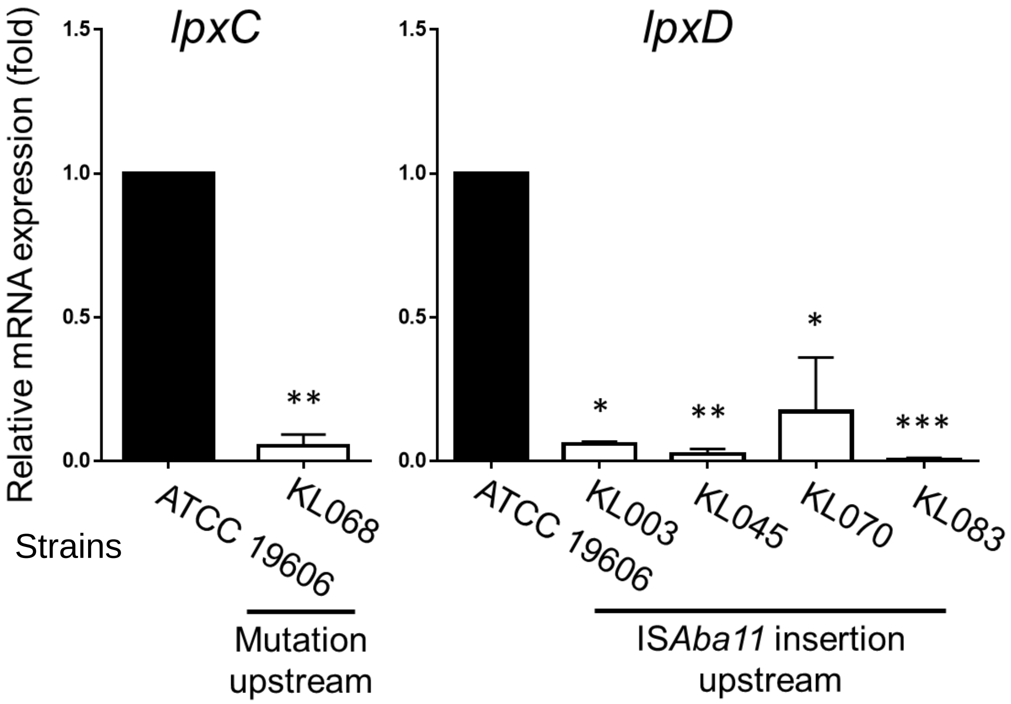

Supplemental
Fig. 2
Kamoshida et al.

A

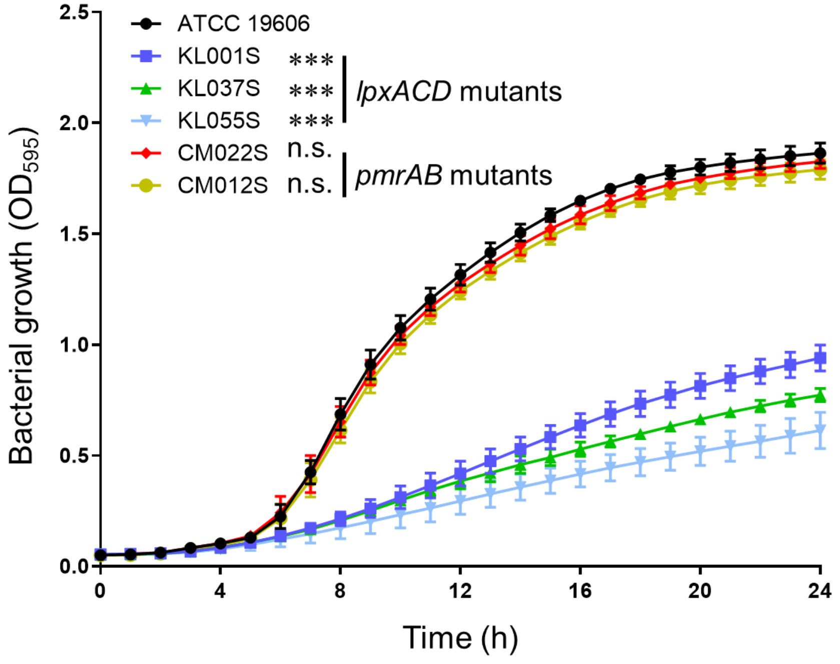

B

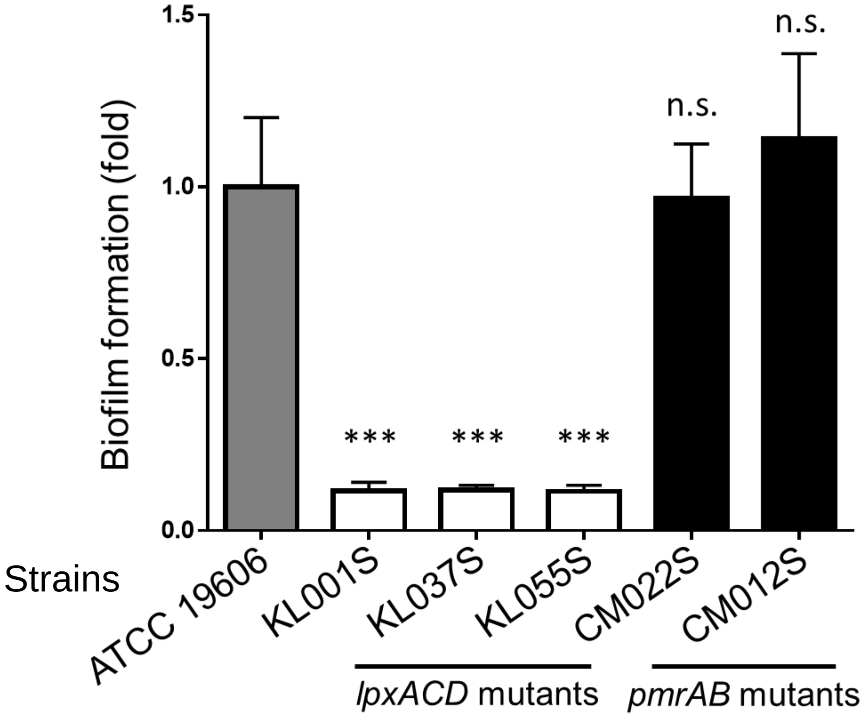

Supplemental
Fig. 3
Kamoshida et al.

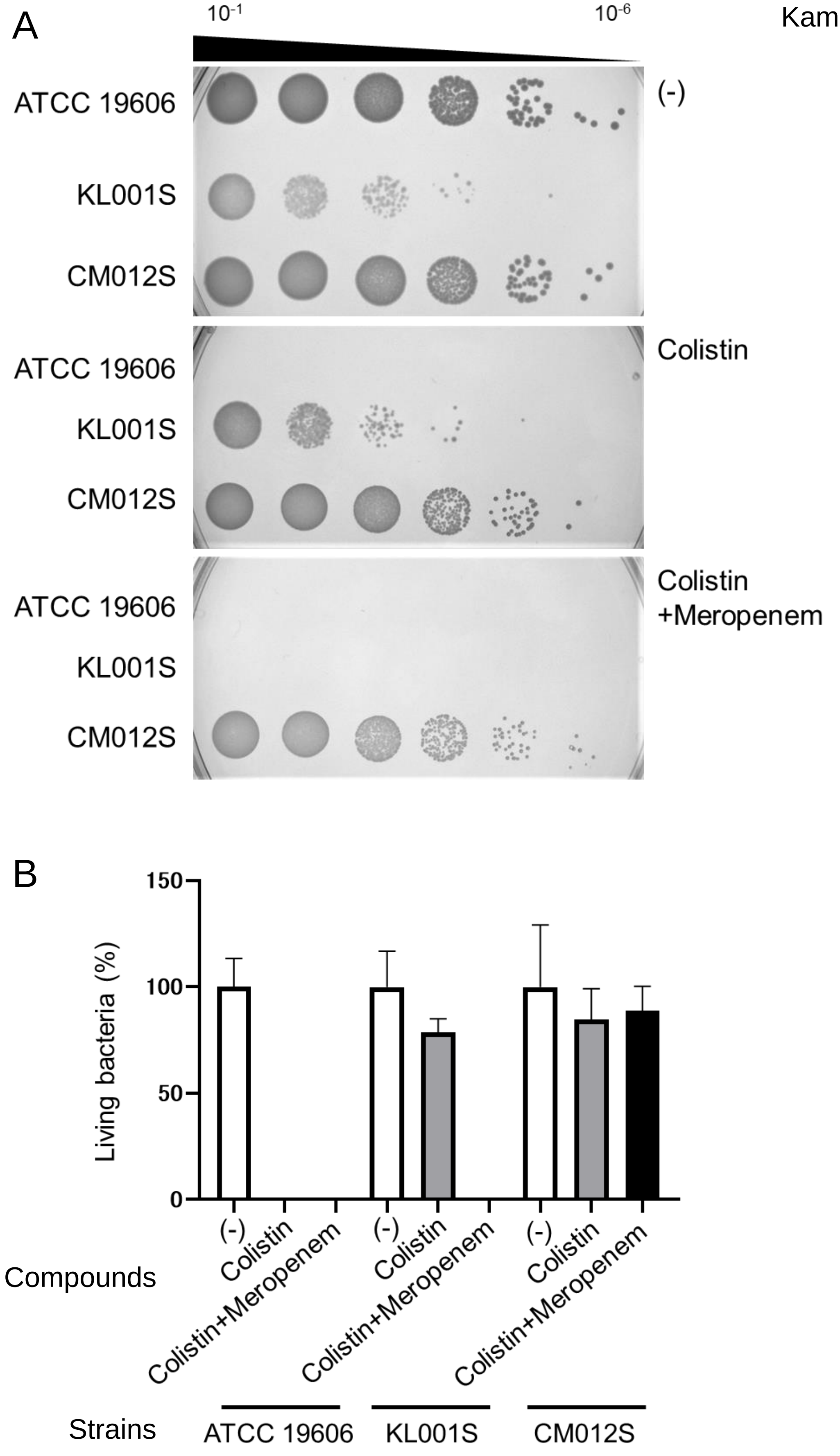

Supplemental
Fig. 4
Kamoshida et al.

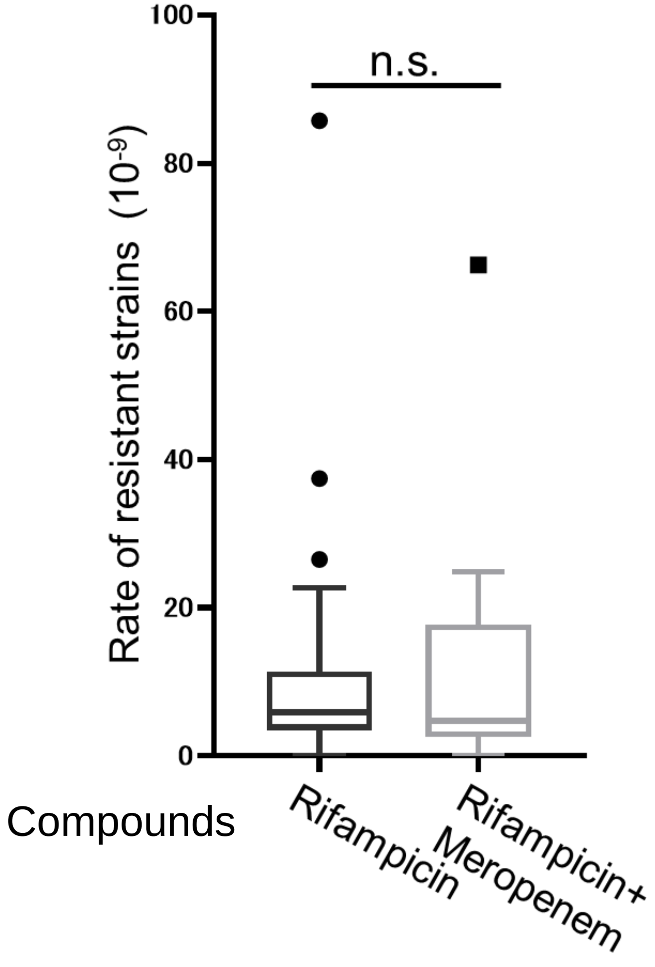

Supplemental TABLE 1 Types of mutations in colistin-resistant strains of *A. baumannii* ATCC 19606.

Supplemental TABLE 2 List of mutations in colistin-resistant strains of *A. baumannii* ATCC 19606.

Supplemental TABLE 3 Minimum inhibitory concentrations (MICs) of colistin in representative *A. baumannii* strains with amino acid substitutions and frameshift mutations in LpxACD.

Supplemental TABLE 4 List of mutations and minimum inhibitory concentrations (MICs) of colistin in *A. baumannii* strains (NCBI BioSample).

Supplemental TABLE 5 List of mutations in colistin-meropenem-resistant strains of *A. baumannii* ATCC 19606.

Supplemental TABLE 6 List of mutations in colistin-ciprofloxacin-resistant strains of *A. baumannii* ATCC 19606.

Supplemental TABLE 7 List of mutations in colistin-resistant and colistin-meropenem-resistant strains of *A. baumannii* ATCC BAA-1605.

Supplemental TABLE 8 Insertion sequences (ISs) in colistin-resistant strains of *A. baumannii* ATCC BAA-1605.

Supplemental FIGURE LEGENDS

Supplemental FIG 1 Expression of *lpxCD* mRNA in colistin-resistant strains with upstream mutations (*lpxC*: KL068; *lpxD*: KL003, KL045, KL070, KL083; and wild-type: ATCC 19606) was analyzed using quantitative reverse transcription-PCR (qRT-PCR). *A. baumannii* was grown in LB broth at 37 °C with shaking until OD_{600} reached 0.75. Total RNA was extracted and reverse-transcribed; RT-qPCR was performed using the intercalator method (TB Green). Data are presented as the mean ± SD (n = 3 per group). Comparison using one-way ANOVA. $***p < 0.001$, $**p < 0.01$, $*p < 0.05$.

Supplemental FIG 2 Growth and biofilm formation of colistin-resistant *A. baumannii* strains. (A) ATCC 19606 (wild-type strain) and representative strains with initial OD_{600} 0.001 were grown in LB broth at 37 °C for 24 h. Absorbance at 595 nm was measured at the indicated time points. Data are presented as the mean ± SD; n = 3 per group compared by two-way ANOVA. n.s., not significant, $***p < 0.001$. (B) The biofilm formation ability of the representative strains was analyzed by the crystal violet staining method. Data are presented as the mean ± SD; n = 3 per group. Compared by one-way ANOVA. n.s., not significant, $***p < 0.001$.

Supplemental FIG 3 Bactericidal activity of antibiotics against LPS-modified and LPS-deficient strains of *A.baumannii* ATCC 19606. (A) Representative images of KL001S and CM012S, LPS-deficient and LPS-modified strains, respectively, and ATCC 19606 as the wild-type strain spotted on each plate. The culture (OD_{600} 1.0) was serially diluted (10^{-1} to 10^{-6}) and 10 μL of each dilution was spotted onto the plates. The upper part of the plate does not contain any antibiotics, the middle part contains 5× MIC (10 μg/mL) of colistin, and the lower part contains 10 μg/mL of colistin and 1/5× MIC (0.1 μg/mL) of meropenem. (B) The CFU obtained on the non-antibiotic plates was used to calculate the percentage of surviving bacteria. Data are presented as the mean ± SD (n = 3 per group).

Supplemental FIG 4 Frequency of resistance to rifampicin alone or in combination with 1/5× MIC (0.1 μg/mL) of meropenem. The culture (0.1 mL of *A. baumannii* ATCC 19606 overnight culture) was plated on LB agar containing 5× MIC (10 μg/mL) of rifampicin with or without 0.1 μg/mL of meropenem. Data are shown as box plots; n = 20 independent assays compared by the Mann–Whitney *U*-test. n.s., not significant.
